# Lecanemab Binds to Transgenic Mouse Model‐Derived Amyloid‐β Fibril Structures Resembling Alzheimer's Disease Type I, Type II and Arctic Folds

**DOI:** 10.1111/nan.70022

**Published:** 2025-06-10

**Authors:** Fernanda S. Peralta Reyes, Simon Sommerhage, Dieter Willbold, Gunnar F. Schröder, Lothar Gremer

**Affiliations:** ^1^ Institut für Physikalische Biologie Heinrich‐Heine‐Universität Düsseldorf Düsseldorf Germany; ^2^ Ernst Ruska‐Centre for Microscopy and Spectroscopy with Electrons, Structural Biology (ER‐C‐3) Forschungszentrum Jülich Jülich Germany; ^3^ Institute of Biological Information Processing, Structural Biochemistry (IBI‐7) Forschungszentrum Jülich Jülich Germany; ^4^ Physics Department Heinrich‐Heine‐Universität Düsseldorf Düsseldorf Germany

**Keywords:** Alzheimer's disease, amyloid‐beta, ARIA‐E, cryo‐EM structures, immunogold‐EM, lecanemab, tg‐mouse models

## Abstract

**Aims:**

Lecanemab, an Alzheimer’s disease US Food and Drug Administration‐approved monoclonal antibody, was previously reported to have a high affinity against intermediately sized amyloid‐β aggregates. Subsequently, it was observed by immunogold labelling that lecanemab can also bind to human type I amyloid‐β fibrils. To determine whether lecanemab binds to amyloid‐β fibril structures other than type I, we analysed its binding capacity to various structurally defined and pathologically relevant amyloid‐β fibrils.

**Methods:**

We performed immunogold labelling with lecanemab on extracted amyloid‐β fibril preparations from six different Alzheimer´s disease mouse models whose structures were previously solved by cryo‐EM and quantified the relative binding affinities of lecanemab to the different fibril polymorphs.

**Results:**

Our results show that lecanemab exhibits high binding affinity to amyloid‐β fibril structures that have a flexible N‐terminus in common, as is the case for type I, type II and murine type III amyloid‐β fibril polymorphs, which resemble or are identical to human structures observed in sporadic and familial cases of Alzheimer’s disease, including a case with the Arctic (E22G) mutation. In contrast, only weak lecanemab binding was observed for murine amyloid‐β fibrils with a fixed and ordered N‐terminus.

**Conclusions:**

These findings may also explain the low incidence of ARIA‐E with lecanemab in clinical trials. This is because human meningeal amyloid‐β fibrils derived from cerebral amyloid angiopathy affected brain tissue also contain a fixed and ordered N‐terminus, most likely preventing lecanemab binding.

**Summary:**

- Lecanemab binds to Aβ fibrils from several Alzheimer's disease tg‐mice whose structures resemble the type I, type II and Arctic folds found in Alzheimer's patients, all of which share a flexible, unstructured N‐terminus.
- Lecanemab is therefore expected to be active against all common familial and sporadic Alzheimer's cases containing these folds.
- Lecanemab binding ability is unaffected by and tolerates the Arctic E22G mutation, at least in type I or Arctic folds.
- Only weak, if any, lecanemab binding was observed to Aβ fibrils derived from tg‐SwDI mice, whose structures DI1, DI2 and DI3 all share structured and fixed N‐termini.
- Since the fixed N‐termini of tg‐SwDI DI1 fibrils and human meningeal Aβ40 fibrils derived from CAA‐affected brain are identical, most likely preventing lecanemab binding, treatment with lecanemab may be less effective or ineffective against CAA, but may explain the reported beneficial low ARIA‐E frequency with this antibody.

## Graphical abstract

Lecanemab binds to Aβ fibrils from several transgenic mouse models of Alzheimer's disease, all of which exhibit flexible, unstructured N‐termini and resemble the type I, type II and Arctic folds found in Alzheimer's patients. Lecanemab is therefore expected to be active against all common familial and sporadic cases of Alzheimer's disease containing these folds. In contrast, only weak binding was observed to Aβ fibrils from tg‐SwDI mice, whose structures DI1, DI2 and DI3 all have structured and fixed N‐termini similar to human meningeal Aβ40 fibrils from CAA‐affected brains, which may explain the reported beneficial low frequency of ARIA‐E with this antibody.

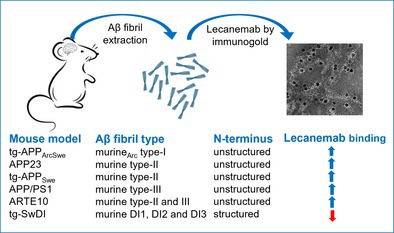

**Abbreviations**
ad
: Alzheimer's disease
ARIA‐E: amyloid‐related imaging abnormalities with oedema
Aβ: amyloid‐beta
AβPP: Aβ protein precursor
CAA: cerebral amyloid angiopathy
cryo‐EM: cryogenic electron microscopy
EM: electron microscopy
FDA: US Food and Drug Administration
PET: positron emission tomography
TEM: transmission electron microscopy
tg‐mouse: transgenic mouse

## 1 Introduction

Alzheimer's disease (ad) is the most common type of dementia and is pathologically associated with the presence of extracellular amyloid‐β (Aβ) plaques and intracellular neurofibrillary tangles. Under pathological conditions, monomeric Aβ aggregates into oligomers, protofibrils, fibrils and eventually plaques [1]. Different therapeutic options intend to target different Aβ species; however, many of them have failed to show clinical efficacy, while others, such as donanemab and lecanemab (a.k.a. BAN2401), are fully approved by the US Food and Drug Administration (FDA) [2, 3]. Additionally, aducanumab has also received an accelerated, conditional FDA approval [2].

mAb158, the murine predecessor antibody of lecanemab, was mainly designed to bind soluble protofibrils rather than mature and insoluble fibrils [4, 5]. Protofibrils have previously been described as soluble intermediate aggregates with a diameter of 6–8 nm that can intertwine to form a structure that can undergo conformational changes, eventually forming mature, insoluble fibrils [4, 5, 6, 7]. Whether protofibrils are indeed separate structural entities, distinct from short soluble fibrils, remains to be clarified. Notwithstanding, ELISA experiments have shown that mAb158 also binds Aβ fibrils, suggesting that the epitope present in protofibrils is also present in fibrillar structures [4, 5]. Nevertheless, one should take into account that mAb158 has no affinity for the Aβ protein precursor (AβPP) and does not bind fibrils from other amyloids [4, 5].

Furthermore, it has been reported that the humanised IgG1 antibody lecanemab can bind with high affinity to soluble protofibrils and only with moderate selectivity to Aβ fibrils when compared to monomeric Aβ [8]. Soluble protofibrils have been portrayed on more than one occasion as the most toxic Aβ species [9], making lecanemab a high‐profile therapeutic option. During a phase 3 clinical trial, lecanemab administration to patients in the early stage of the disease showed decreased amyloid levels in the brain and a moderate reduction of cognitive decline when compared to placebo [8]. When interpreting the data regarding amyloid levels in the brain, it is worth highlighting that lecanemab does not interfere with the positron emission tomography (PET) radioligand [^11^C]‐Pittsburgh compound B when binding to Aβ deposits, as both have different binding sites [10].

Although it was reported that lecanemab does not have a high binding affinity for fibrils [8], it has been shown by immunohistochemistry that lecanemab also stains Aβ plaques [11]. Additionally, it was portrayed by immunogold‐electron microscopy (immunogold‐EM) that lecanemab can indeed bind Aβ fibrils that were observed in ultracentrifugal supernatants of aqueous extracts from the human brain parenchyma [12], as shown for two samples containing mainly Aβ fibrils denominated as type I [12], observed in sporadic and familial ad cases [13, 14]. Whether lecanemab can also bind to other pathologically relevant types of Aβ polymorphs remains to be elucidated.

Therefore, we analysed the binding competency of lecanemab by immunogold‐EM on various ex vivo Aβ fibril polymorphs. These Aβ fibrils were derived from brain samples of six common pre‐clinical ad transgenic mouse (tg‐mouse) models, whose structures have been recently solved by cryo‐EM [15]. Our selection includes the tg‐APP_ArcSwe_ tg‐mouse model, which was used in the pre‐clinical evaluation of lecanemab [16] and is, so far, the only model whose Aβ fibrils resemble the human type I Aβ polymorph. Additionally, ad tg‐mouse models that exhibit fibrils of the human type II polymorph, mainly observed in familial ad cases and other conditions, as well as tg‐mouse models resembling the human Arctic fibril fold, and finally, a tg‐mouse model with other novel Aβ structures were assessed [15].

## 2 Materials and Methods

### 2.1 Animals

The ex vivo Aβ fibril sample preparations analysed in the present study were previously used to solve their cryo‐EM structures [15] and were isolated from the following mouse models: APP/PS1 (APPswe/PSEN1delE9) (heterozygous; *n* = 1 (male); 33 months old) on a C57BL/6;C3H background. ARTE10 (homozygous; *n* = 1 (female); 24 months old) on a C57Bl/6 background, which was a gift from Taconic Biosciences. Tg‐SwDI (heterozygous; *n* = 1 (male); 29 months old) on a C57BL/6 background, which is not only used as a model for AD but also for cerebral amyloid angiopathy (CAA). APP23 (heterozygous; *n* = 1 (male); 21 months old) on a C56BL/6 background. Tg‐APP_ArcSwe_ (heterozygous; *n* = 1 (male); 18 months old) and tg‐APP_Swe_ (heterozygous; *n* = 1 (male); 22 months old), both on a C57BL/6 background.

### 2.2 Aβ Fibril Extraction

Aβ fibril extraction was done using sarkosyl solubilisation [14, 15]. Between 0.4 and 0.6 g of non‐fixed brain tissue from six different ad tg‐mouse models was snap‐frozen in −80 °C cold isopentane and stored at −80 °C. The tissue was then thawed and physically homogenised in a 20‐fold volume (w/v) of extraction buffer (10 mM Tris–HCl, pH 7.5, 0.8 M NaCl, 10% sucrose, 1 mM EGTA) using a Dounce glass tissue grinder. 10% aqueous sarkosyl (Sigma‐Aldrich) was added to bring the brain homogenate to a final sarkosyl concentration of 2%. The sample was mixed thoroughly by pipetting up and down 30 times before incubation at 37 °C for 1 h. The homogenate was then centrifuged at 10,000×*g* in a tabletop centrifuge at 4 °C for 10 min. The pellet was discarded, and the supernatant was ultracentrifuged at 100,000x*g* at 4 °C for 1 h (Beckman Coulter Optima MAX‐XP, TLA55 fixed‐angle rotor). The resulting supernatant was discarded, and the pellet was resuspended and mixed with extraction buffer (1 mL·g^−1^ original tissue mass) before low‐speed centrifugation at 5000×*g* at 4 °C for 5 min. Afterwards, the supernatant was threefold diluted in dilution buffer (50 mM Tris–HCl, pH 7.5, 0.15 M NaCl, 10% sucrose, 0.2% sarkosyl) and ultracentrifuged once more at 100,000×*g* at 4 °C for 30 min. The final Aβ fibril‐rich pellet was resuspended (100 μL·g^−1^ original tissue mass) in resuspension buffer (20 mM Tris–HCl, pH 7.4, 50 mM NaCl), frozen in liquid nitrogen and stored at −80 °C for further use.

### 2.3 Immunogold Labelling

Immunogold labelling was performed according to the protocol of Gulati et al. [17]. In brief, 300‐mesh carbon‐coated copper grids (EM Sciences, ECF300‐CU) were glow discharged with a PELCO easiGlow Glow Discharge Cleaning System. Three microliters samples of extracted Aβ fibril suspension were incubated for 2 min on the grid's surface and excess liquid was blotted with filter paper afterwards. The grid was then placed on top of a 15 μL H_2_O droplet on parafilm for 1 min and blotted. Afterwards, the grid was transferred to a 15 μL droplet of blocking buffer (99 mL PBS, pH 7.4, 100 μL Tween‐20, 1 mL 30% IgG‐free bovine serum albumin) inside a humidifying chamber and incubated for 15 min. After blotting, the grid was transferred to a 15 μL droplet of lecanemab primary antibody diluted in blocking buffer to 2 μg·ml^−1^ for 1–2 h and blotted once more. The grid was washed five times by incubating it in 15 μL droplets of washing buffer (100 mL PBS, pH 7.4, 100 μL Tween‐20, 100 μL 30% IgG‐free bovine serum albumin) for 3 min and blotting with filter paper after each wash. The grid was then transferred to a 10 nm gold‐conjugated goat anti‐human secondary antibody (Abcam) diluted 1:20 in a droplet of blocking buffer for 1 h. The grid was washed five times with washing buffer and three times with H_2_O as described above. The grid was then transferred to a 15 μL droplet of 1% uranyl acetate for 1 min, blotted and air‐dried. The prepared grids were examined on a Talos L120C G2 transmission electron microscope (Thermo Fisher Scientific) operated at 120 kV (LaB6/Denka). For each sample, a dataset of high magnification (57,000‐fold) micrographs was collected on a 4 k × 4 k Ceta 16 M CEMOS camera using the Thermo Scientific Velox user interface: 125 micrographs for tg‐APP_ArcSwe_, 101 for APP23, 112 for tg‐APP_Swe_, 101 for APP/PS1, 130 for ARTE10 and 160 for tg‐SwDI. The TEM images were collected manually in specimen areas of sufficient quality.

### 2.4 Micrograph Annotation, Calculation of Gold Particle‐Fibril Distances and Lecanemab Binding Scores

Coordinates of gold particles and Aβ fibrils were manually annotated in micrographs using Napari [18]. Gold particles were represented by their centre coordinates relative to the micrograph and fibrils by the coordinates of their start and end points. Only fibrils that were sufficiently separated from each other were selected to exclude those fibrils that were sterically inaccessible for antibody binding due to other attached fibrils. From this data, the distances of all gold particles to all fibrils were calculated using the shortest distance between a point and a line defined by the fibril coordinates. For particles at the fibril ends, just the Euclidean distance to the corresponding end was calculated. The number of bound gold particles was then determined for each fibril. A gold particle was defined as bound to a certain fibril, if the distance was below a threshold, determined by the gold particle radius r, the size of the primary and secondary antibody a and the width of the fibril d: r+a+d/2. The gold particle radius and the fibril width were measured manually on the micrographs with r=5 nm and d=17 nm, while the typical length of a primary and secondary antibody complex a=30 nm was taken from literature [19]. If the distances between a particle and multiple fibrils were below the threshold, the closest fibril was selected to ensure that each bound particle was assigned to only one fibril. To define a comparable binding score, the number of bound gold particles for each fibril was divided by the corresponding fibril length.

### 2.5 Statistical Significance Testing

Statistical significance between the gold particles per fibril length scores of the different samples was assessed using a two‐step approach. First, an omnibus test determined overall differences across distributions: either one‐way ANOVA (parametric) for normally distributed data or the Kruskal–Wallis *H* test (non‐parametric) when normality assumptions were violated (assessed via the Shapiro–Wilk test). For significant omnibus results (*p* < 0.05), post hoc pairwise comparisons were conducted using either Tukey's honest significant difference test (following ANOVA) or Mann–Whitney *U* tests with Bonferroni correction (following Kruskal–Wallis). When comparing to a reference group, only pairwise comparisons involving the reference were performed, with Bonferroni correction adjusted accordingly. Significance levels were denoted using standard asterisk notation (*p < 0.05, **p < 0.01, ***p < 0.001). Importantly, all statistical tests were performed on complete datasets, while visualisations excluded outliers (defined as values beyond 1.5 × IQR from quartiles) to improve clarity.

### 2.6 Data and Code Availability

The distance calculation, statistical testing and subsequent generation of plots were implemented in Python utilising the following open‐source libraries: Numpy, Pandas, Matplotlib, Seaborn, Scipy and Statsmodels [20, 21, 22, 23, 24, 25, 26, 27]. The corresponding Jupyter notebook and annotated coordinate data were uploaded to a repository on GitHub: https://github.com/sim‐som/filament_particle_co_localization.

All collected TEM images are publicly accessible in the following Zenodo repository: https://doi.org/10.5281/zenodo.15232810.

## 3 Results and Discussion

**FIGURE 1:**
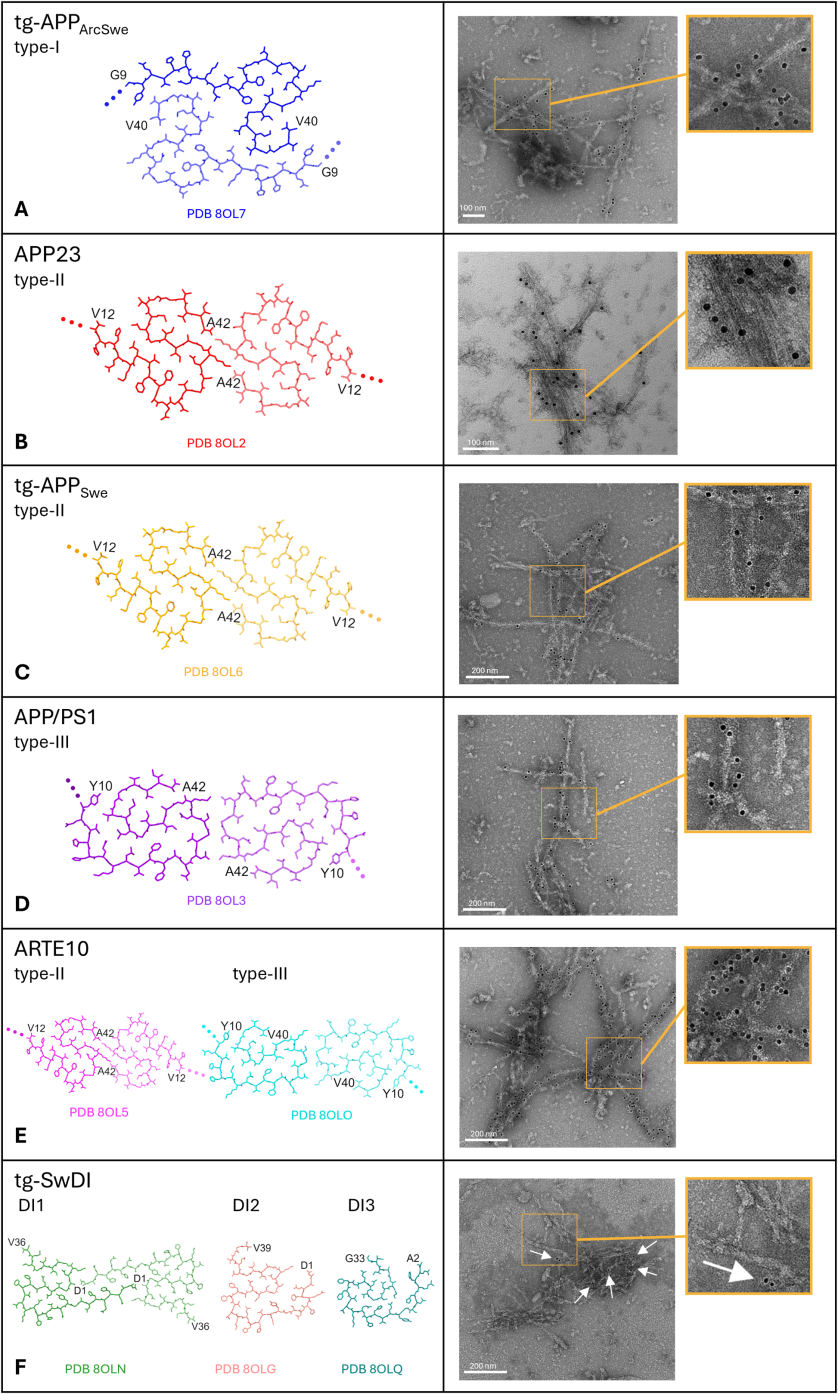
Lecanemab immunogold‐labelling of Aβ fibrils with different molecular structures. Previously solved cryo‐EM structures of Aβ fibrils from different ad mouse models [15] (left). Immunogold TEM images of extracted Aβ fibrils with lecanemab as the primary antibody (right). Only a few gold particles (white arrows) labelled the Aβ fibrils from the tg‐SwDI mouse model (F, right panel), which exhibit fixed N‐termini (F, left panel). In contrast, the Aβ fibril folds of all other mouse models have unstructured, flexible N‐termini instead (A–E, left panels; dotted lines).

First, we tested by immunogold‐EM labelling whether lecanemab can bind ex vivo murine type I Aβ fibrils obtained from tg‐APP_ArcSwe_ mouse brain tissue. The fibrils were incubated with lecanemab as the primary antibody, then reacted with the secondary antibody with conjugated 10 nm gold nanoparticles, followed by negative‐staining with uranyl acetate. The TEM electron micrographs reveal a specific fibril decoration with the electron‐dense 10 nm gold nanoparticles (Figure 1A), indicating specific lecanemab binding to type I Aβ fibrils from tg‐APP_ArcSwe_ mouse brain. Of note, the tg‐APP_ArcSwe_ mouse model was used for the pre‐clinical validation of lecanemab [16] and is the only tg‐ad mouse model up to date that resembles the human type I Aβ fibril polymorph [15], present in sporadic [14] and familial ad cases [13], including Down's syndrome [28]. Although murine and human type I Aβ fibrils show subtle differences due to the Arctic (E22G) mutation in the tg‐APP_ArcSwe_ mice, their overall fold, side‐chain orientation and fibril surface are conserved. Indeed, previous findings have shown that lecanemab binds to human ex vivo type I Aβ fibrils [12] that do not carry any mutation, indicating that the presence or absence of the E22G mutation does not affect lecanemab binding.

In familial ad and other conditions, another Aβ fibril fold, named type II was identified as well [14, 28]. Therefore, we were interested in whether lecanemab can also bind and detect this fibril polymorph by using type II Aβ fibrils extracted from brains from tg‐APP_Swe_ or APP23 tg‐mice, respectively [15]. After performing immunogold‐EM, a clear gold labelling of the Aβ fibrils from both mouse models was observed (Figure 1B,C), indicating specific lecanemab binding to type II fibrils as well. Considering that the 3D structures of type II Aβ fibrils in humans and mice are identical down to atomic details [15], we expect lecanemab to be effective against patients with ad pathologies involving Aβ type II fibrils as well.

Furthermore, another Aβ fibril fold referred to as murine type III was observed in Aβ fibril preparations from brain tissue of APP/PS1 and ARTE10 tg‐mice, the latter together with Aβ type II fibrils [15]. Notably, the murine Aβ type III fibril structure with its nonmutated Aβ42 sequence is highly similar to a protofilament pair involving protofilaments A and B from a tetrameric human Arctic (E22G) Aβ fibril fold [29].

After immunogold staining, the electron micrographs show that lecanemab also binds and recognises type III fibrils present in APP/PS1 tg‐mice (Figure 1D). In addition, fibril preparations derived from ARTE10 tg‐mice, which displayed both type II and type III fibrils (Figure 1E), also show specific lecanemab binding, as expected.

Our previous findings demonstrate a structural similarity between the murine Aβ type III fold and the human Arctic fold [15, 29]. Therefore, our findings suggest that lecanemab may also be effective in ad patients exhibiting this fold, whether in its non‐mutated form (as in APP/PS1 and ARTE10 tg‐mice [15]) or in the E22G‐mutated form (as in ad patients with the Arctic mutation [29]).

Other novel Aβ polymorphs designated DI1, DI2 and DI3, with DI1 as the most abundant, were observed in Aβ fibril preparations from brains of tg‐SwDI mice [15], which serves as a model for both ad and CAA. In contrast to the type I, type II and murine Aβ type III folds, which all have in common a flexible N‐terminus, all three resolved tg‐SwDI folds exhibit well‐ordered and fixed N‐termini [15] (Figure 1F). Analysis of lecanemab's binding capability to SwDI tg‐mice derived Aβ fibrils by immunogold‐EM revealed only few gold particles bound to SwDI fibrils, which indicates a weak or negligible lecanemab binding (Figure 1F).

**FIGURE 2:**
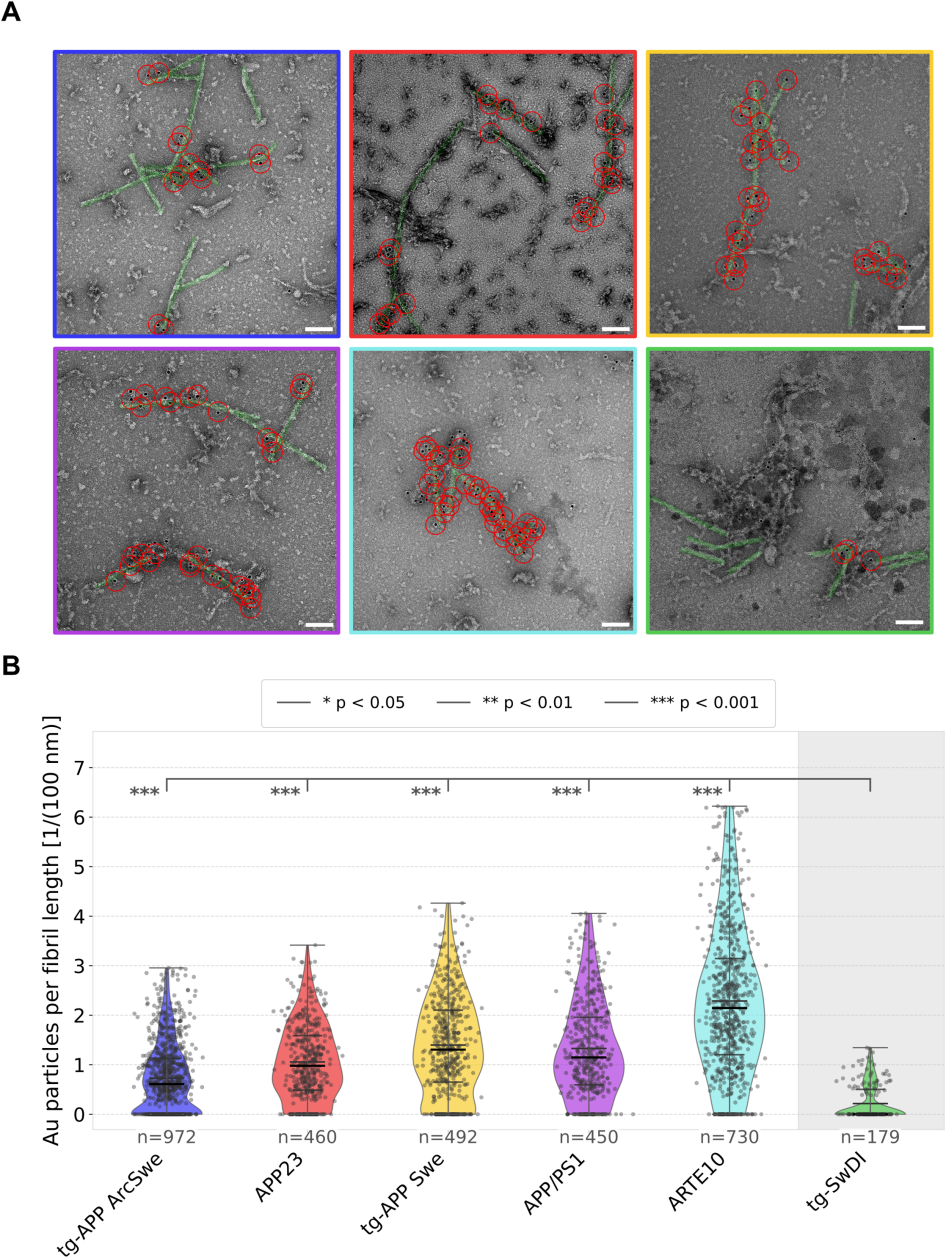
Gold nanoparticle co‐localization and distribution analysis of lecanemab immunogold. (A) Representative TEM micrographs (scale bars 100 nm) of immunogold‐labelled samples with annotated Aβ fibrils highlighted in green and annotated antibody‐bound gold particles encircled in red. The radius of each red circle represents the combined radius of a gold particle plus the expected spatial extent of the primary and secondary antibodies. In the analysis, a gold particle antibody complex is considered bound to the fibrils if the red circle and the green fibril region overlap. For each sample, at least ~ 100 micrographs were collected and analysed. (B) Comparison of the gold particles per fibril distribution across samples. In the violin plots, scattered dots represent individual measurements, while the violin shape was drawn using a kernel density estimate of the underlying distribution. The sample mean is denoted by a bold dark line, while quartiles are represented by thinner lines. Overall, the analysis shows a significant difference between the binding affinity to tg‐SwDI fibrils (rightmost column) and the other samples. Statistical significance testing was performed comparing each group to the tg‐SwDI data, with *p* values indicated for each comparison.

To further quantify the fibril type‐dependent differences in relative binding affinity initially observed by immunogold labelling (Figure 1), TEM data sets with at least 100 micrographs were collected for each immunogold‐labelled sample. After manually annotating the positions of fibrils and gold nanoparticles, the number of bound particles per fibril length was calculated to serve as a measure of relative binding affinity. A particle was considered bound if the shortest distance between its centre and the fibril axis was smaller than a threshold of ~ 44 nm defined by the fibril diameter (8.5 nm), the gold particle radius (5 nm) and the size of the primary‐secondary antibody complex (30 nm) (Figure 2A). The number of annotated fibrils per data set, i.e., the number of particles per fibril length measurements was sufficient to allow a meaningful comparison of the fibril types' binding affinities to lecanemab (Figure 2B). Indeed, statistical analysis showed that lecanemab binding to tg‐SwDI fibrils was significantly (*p* < 0.001) weaker compared to all other tg‐mouse Aβ fibril samples (Figure 2B).

Considering that lecanemab's binding site is reported on the N‐terminus (between residues 1–16) [30], the results indicate that the N‐terminus needs to be flexible and non‐structured to act as an efficient lecanemab binding epitope. This is evident by the higher degree of observed lecanemab binding to the type I, type II and the murine type III Aβ folds (Figure 1A–E, Figure 2) in comparison to the tg‐SwDI folds (Figure 1F, Figure 2). Interestingly, when compared to immunogold‐labelling using NAB228 as primary antibody, which also binds to the N‐terminus (1–11), a similar pattern was observed [15].

**FIGURE 3:**
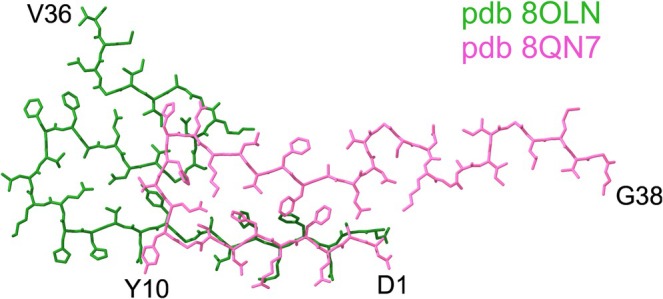
Structural similarity of the N‐termini of DI1 Aβ fibrils from tg‐SwDI mice and Aβ40 fibrils from the leptomeninges of human brain tissue. Overlay of the DI1 Aβ fibril structure from tg‐SwDI mice (green; pdb 8OLN) with the cryo‐EM structure of Aβ40 fibrils extracted from the leptomeninges of human brain tissue from a patient with Alzheimer's disease (pink, pdb 8QN7) showing the similarities between their structured N‐termini.

Even though the tg‐SwDI folds are unlikely to be present in humans due to the double, Dutch (E22Q) and Iowa mutations (D23N) [11], the N‐termini of tg‐SwDI DI1 and Aβ40 fibrils extracted from the human meninges [15, 31, 32] are structurally highly similar [15]. (Figure 3). Therefore, lecanemab may have less binding affinity to CAA cases, as was also confirmed in a previous study by Söderberg and colleagues [33]. There it was also observed that lecanemab has a relatively low frequency of amyloid‐related imaging abnormalities with oedema (ARIA‐E) (12.6%) when compared to other antibodies such as aducanumab, bapineuzumab, donanemab and gantenerumab, which have higher ARIA‐E frequencies (25–35%) and higher binding affinity to CAA fibrils [33].

In conclusion, our results show that lecanemab is expected to be active against all common familial and sporadic ad cases containing type I, type II or the Arctic fold, or mixtures of them, all having a flexible N‐terminus in common. Since lecanemab binds the type I Aβ fibril fold in its non‐mutated state (humans) [12] as well as in the E22G‐mutated state (tg‐APP_ArcSwe_ mice), it is conceivable that lecanemab may also bind to other Aβ fibrils carrying other E22 or neighbouring mutations (i.e., Flemish A21G, Dutch E22Q, Italian E22K, Iowa D23N), as long as the fibril fold with a flexible N‐terminus is maintained. This principle may not be restricted to type I fibrils, as the type III fold (non‐mutated in mice) and the human Arctic E22G fold are also structurally similar. Further research may focus on a detailed structural characterisation of the lecanemab binding modes to the various AD‐relevant Aβ fibril folds.

## Author Contributions

F.S.P.R. conducted the ex vivo Aβ fibril preparations, their lecanemab immunogold‐labelling, negative‐stain EM, collection of EM micrographs and immunogold particle co‐localisation. S.S. performed the statistical analysis regarding gold particle per fibril distribution. D.W., L.G. and G.F.S. supervised the project. F.S.P.R. and L.G. wrote the original manuscript draft, and F.S.P.R., S.S., D.W., L.G. and G.F.S. edited and reviewed the final manuscript.

## Ethics Statement

Experiments that were performed on the APP/PS1, ARTE10, tg‐SwDI and APP23 AD mouse models were conducted in line with the German Law on the protection of animals (TierSchG §§7–9). APP/PS1 mice breeding was validated by a local ethics committee (Landesamt für Natur, Umwelt und Verbraucherschutz Nordrhein‐Westfalen (LANUV), Az: 84–02.04.2019.A304). APP/PS1 and tg‐SwDI mouse lines were acquired from the Jackson Lab (JAX MMRRC Stock no. 034829 or JAX MMRRC Stock no. 034843). Breeding of tg‐APP_ArcSwe_ and tg‐APP_Swe_ mouse models was done under the ethical permit 5.8.18–20,401/20, approved by the Uppsala County Animal Ethics Board. All mice were treated under controlled conditions at 22 °C, 12:12 h light/dark cycle, 54% humidity, as well as food and water ad libitum.

## Conflicts of Interest

D.W. is a founder and shareholder of the companies Priavoid and Attyloid and a member of their supervisory boards. This did not influence the interpretation of the data. All other authors declare no competing interests.

## Data Availability

The data that support the findings of this study are openly available in Zenodo at https://doi.org/10.5281/zenodo.15232810, reference number DOI: 10.5281/zenodo.15232810.

## References

[1] H. Hampel , J. Hardy , K. Blennow , et al., “The Amyloid‐β Pathway in Alzheimer's Disease,” Molecular Psychiatry 26, no. 10 (2021): 5481–5503, 10.1038/s41380-021-01249-0. 34456336 PMC8758495

[2] J. Zhang , Y. Zhang , J. Wang , Y. Xia , J. Zhang , and L. Chen , “Recent Advances in Alzheimer's Disease: Mechanisms, Clinical Trials and New Drug Development Strategies,” Signal Transduction and Targeted Therapy 9, no. 1 (2024): 211, 10.1038/s41392-024-01911-3. 39174535 PMC11344989

[3] C. Kang , “Donanemab: First Approval,” Drugs 84 (2024): 1313–1318, 10.1007/s40265-024-02087-4. 39237715

[4] H. Englund , D. Sehlin , A.‐S. Johansson , et al., “Sensitive ELISA Detection of Amyloid‐Beta Protofibrils in Biological Samples,” Journal of Neurochemistry 103, no. 1 (2007): 334–345, 10.1111/j.1471-4159.2007.04759.x. 17623042

[5] D. Sehlin , X. T. Fang , L. Cato , G. Antoni , L. Lannfelt , and S. Syvänen , “Antibody‐Based PET Imaging of Amyloid Beta in Mouse Models of Alzheimer's Disease,” Nature Communications 7 (2016): 10759, 10.1038/ncomms10759. PMC4762893 26892305

[6] J. C. Rochet and P. T. Lansbury , “Amyloid Fibrillogenesis: Themes and Variations,” Current Opinion in Structural Biology 10, no. 1 (2000): 60–68, 10.1016/s0959-440x(99)00049-4. 10679462

[7] A.‐S. Johansson , F. Berglind‐Dehlin , G. Karlsson , K. Edwards , P. Gellerfors , and L. Lannfelt , “Physiochemical Characterization of the Alzheimer's Disease‐Related Peptides A Beta 1‐42Arctic and A Beta 1‐42wt,” FEBS Journal 273, no. 12 (2006): 2618–2630, 10.1111/j.1742-4658.2006.05263.x. 16817891

[8] C. H. van Dyck , C. J. Swanson , P. Aisen , et al., “Lecanemab in Early Alzheimer's Disease,” New England Journal of Medicine 388, no. 1 (2023): 9–21, 10.1056/NEJMoa2212948. 36449413

[9] D. J. Rinauro , F. Chiti , M. Vendruscolo , and R. Limbocker , “Misfolded Protein Oligomers: Mechanisms of Formation, Cytotoxic Effects, and Pharmacological Approaches Against Protein Misfolding Diseases,” Molecular Neurodegeneration 19, no. 1 (2024): 20, 10.1186/s13024-023-00651-2. 38378578 PMC10877934

[10] M. Xiong , A. Dahlén , S. Roshanbin , et al., “Antibody Engagement With Amyloid‐Beta Does Not Inhibit 11CPib Binding for PET Imaging,” Journal of Neurochemistry 168, no. 9 (2024): 2601–2610, 10.1111/jnc.16127. 38721627

[11] M. Johannesson , C. Sahlin , L. Söderberg , et al., “Elevated Soluble Amyloid Beta Protofibrils in Down Syndrome and Alzheimer's Disease,” Molecular and Cellular Neuroscience 114 (2021): 103641, 10.1016/j.mcn.2021.103641. 34091073

[12] A. M. Stern , Y. Yang , S. Jin , et al., “Abundant Aβ Fibrils in Ultracentrifugal Supernatants of Aqueous Extracts From Alzheimer's Disease Brains,” Neuron 111, no. 13 (2023): 2012–2020, 10.1016/j.neuron.2023.04.007. 37167969 PMC10330525

[13] M. R. Hoq , A. Fernandez , F. S. Vago , et al., “Cryo‐EM Structures of Cotton Wool Plaques' Amyloid β and of Tau Filaments in Dominantly Inherited Alzheimer Disease,” Acta Neuropathologica 148, no. 1 (2024): 20, 10.1007/s00401-024-02786-y. 39147931 PMC11327195

[14] Y. Yang , D. Arseni , W. Zhang , et al., “Cryo‐EM Structures of Amyloid‐β 42 Filaments From Human Brains,” Science 375, no. 6577 (2022): 167–172, 10.1126/science.abm7285. 35025654 PMC7612234

[15] M. Zielinski , F. S. Peralta Reyes , L. Gremer , et al., “Cryo‐EM of Aβ Fibrils From Mouse Models Find tg‐APPArcSwe Fibrils Resemble Those Found in Patients With Sporadic Alzheimer's Disease,” Nature Neuroscience 26, no. 12 (2023): 2073–2080, 10.1038/s41593-023-01484-4. 37973869 PMC10689242

[16] S. Tucker , C. Möller , K. Tegerstedt , et al., “The Murine Version of BAN2401 (mAb158) Selectively Reduces Amyloid‐β Protofibrils in Brain and Cerebrospinal Fluid of tg‐ArcSwe Mice,” Journal of Alzheimer's Disease 43, no. 2 (2015): 575–588, 10.3233/JAD-140741. 25096615

[17] N. M. Gulati , U. Torian , J. R. Gallagher , and A. K. Harris , “Immunoelectron Microscopy of Viral Antigens,” Current Protocols in Microbiology 53, no. 1 (2019): e86, 10.1002/cpmc.86. 31219685 PMC6588173

[18] N. Sofroniew , T. Lambert , G. Bokota , et al., Napari: A Multi‐Dimensional Image Viewer for Python. v0.5.6 (Zenodo, 2025), 10.5281/zenodo.3555620.

[19] R. Hermann , P. Walther , and M. Müller , “Immunogold Labeling in Scanning Electron Microscopy,” Histochemistry and Cell Biology 106, no. 1 (1996): 31–39, 10.1007/BF02473200. 8858365

[20] Proceedings of the 9th Python in Science Conference (2010), 10.25080/Majora-92bf1922-012.

[21] C. R. Harris , K. J. Millman , S. J. van der Walt , et al., “Array Programming With NumPy,” Nature 585, no. 7825 (2020): 357–362, 10.1038/s41586-020-2649-2. 32939066 PMC7759461

[22] J. D. Hunter , “Matplotlib: A 2D Graphics Environment,” Computing in Science & Engineering 9, no. 3 (2007): 90–95, 10.1109/MCSE.2007.55.

[23] W. McKinney , “Data Structures for Statistical Computing in Python,” in Proceedings of the 9th Python in Science Conference (2010), 56–61, 10.25080/Majora-92bf1922-00a.

[24] S. Seabold and J. Perktold , “Statsmodels: Econometric and Statistical Modeling With Python,” in *Proceedings of the* *9th Python in Science Conference* (2010), 92–96, 10.25080/Majora-92bf1922-011.

[25] The Pandas Development Team , Pandas‐Dev/Pandas: Pandas. v2.2.3 (*Zenodo* 2024), 10.5281/zenodo.13819579.

[26] P. Virtanen , R. Gommers , T. E. Oliphant , et al., “SciPy 1.0: Fundamental Algorithms for Scientific Computing in Python,” Nature Methods 17 (2020): 261–272, 10.1038/s41592-019-0686-2. 32015543 PMC7056644

[27] M. L. Waskom , “Seaborn: Statistical Data Visualization,” Journal of Open Source Software 6, no. 60 (2021): 3021, 10.21105/joss.03021.

[28] A. Fernandez , M. R. Hoq , G. I. Hallinan , et al., “Cryo‐EM Structures of Amyloid‐β and Tau Filaments in Down Syndrome,” Nature Structural & Molecular Biology 31, no. 6 (2024): 903–909, 10.1038/s41594-024-01252-3. PMC11189299 38553642

[29] Y. Yang , W. Zhang , A. G. Murzin , et al., “Cryo‐EM Structures of Amyloid‐β Filaments With the Arctic Mutation (E22G) From Human and Mouse Brains,” Acta Neuropathologica 145, no. 3 (2023): 325–333, 10.1007/s00401-022-02533-1. 36611124 PMC9925504

[30] S. S. Plotkin and N. R. Cashman , “Passive Immunotherapies Targeting Aβ and Tau in Alzheimer's Disease,” Neurobiology of Disease 144 (2020): 105010, 10.1016/j.nbd.2020.105010. 32682954 PMC7365083

[31] Y. Yang , A. G. Murzin , S. Peak‐Chew , et al., “Cryo‐EM Structures of Aβ40 Filaments From the Leptomeninges of Individuals With Alzheimer's Disease and Cerebral Amyloid Angiopathy,” Acta Neuropathologica Communications 11, no. 1 (2023): 191, 10.1186/s40478-023-01694-8. 38049918 PMC10694933

[32] M. Kollmer , W. Close , L. Funk , et al., “Cryo‐EM Structure and Polymorphism of Aβ Amyloid Fibrils Purified From Alzheimer's Brain Tissue,” Nature Communications 10, no. 1 (2019): 4760, 10.1038/s41467-019-12683-8. PMC6820800 31664019

[33] L. Söderberg , M. Johannesson , E. Gkanatsiou , et al., “Amyloid‐Beta Antibody Binding to Cerebral Amyloid Angiopathy Fibrils and Risk for Amyloid‐Related Imaging Abnormalities,” Scientific Reports 14, no. 1 (2024): 10868, 10.1038/s41598-024-61691-2. 38740836 PMC11091209

